# A Case Report of Acquired Hepatocerebral Degeneration Mimicking Parkinson’s Disease in Presumed Metabolic Dysfunction-Associated Steatotic Liver Disease (MASLD)-Related Cirrhosis

**DOI:** 10.7759/cureus.114216

**Published:** 2026-08-09

**Authors:** Fahmida Tithi, Arundhathy Krishna, Nazia Binte Salam, Juan Pedro Simon-Turriate, Benjamin Young

**Affiliations:** 1 General Internal Medicine, East Surrey Hospital, Surrey and Sussex Healthcare NHS Trust, Redhill, GBR; 2 Cardiology, East Surrey Hospital, Surrey and Sussex Healthcare NHS Trust, Redhill, GBR; 3 Internal Medicine, East Surrey Hospital, Surrey and Sussex Healthcare NHS Trust, Redhill, GBR; 4 Geriatrics, East Surrey Hospital, Surrey and Sussex Healthcare NHS Trust, Redhill, GBR

**Keywords:** atypical parkinsonism, cirrhosis of the liver, cognitive impairnment, metabolic dysfunction-associated steatotic liver disease (masld), metabolic encephalopathy, porto-systemic shunt

## Abstract

Acquired hepatocerebral degeneration (AHD) is a rare chronic neurological syndrome associated with advanced liver disease and portosystemic shunting, characterized by neuropsychiatric and extrapyramidal manifestations resembling those seen in hepatolenticular degeneration (Wilson's disease). We report a rare case of AHD presenting as rapidly progressive parkinsonism with cognitive decline. A 64-year-old man with poorly controlled type 2 diabetes mellitus and obesity developed progressive tremor, gait instability, recurrent falls, and worsening cognition. Initial assessment suggested benign tremor and early Parkinson’s disease; however, further evaluation demonstrated cirrhosis with portal hypertension, hyperammonaemia, and hepatic encephalopathy. Brain imaging revealed characteristic basal ganglia abnormalities consistent with AHD. This case highlights the importance of considering underlying liver disease in patients presenting with atypical parkinsonism and cognitive deterioration and also emphasizes the limited effectiveness of conventional Parkinson’s disease therapies in this setting.

## Introduction

Acquired hepatocerebral degeneration (AHD) is a rare chronic neurological syndrome characterized by parkinsonism, ataxia, other movement disorders, and neuropsychiatric and cognitive impairment in patients with chronic liver disease, particularly those with portosystemic shunting [1]. Unlike hepatic encephalopathy, which typically presents with fluctuating and potentially reversible neuropsychiatric manifestations, AHD is characterized by persistent neurological deficits and is thought to result from chronic exposure to neurotoxic substances rather than acute metabolic disturbances [2]. Portosystemic shunting bypasses normal hepatic detoxification, allowing neurotoxins, particularly manganese, to accumulate within the basal ganglia and contribute to progressive neurological dysfunction [3]. The syndrome was first described by Van Woerkem in 1914 [4] but gained wider recognition after the seminal report by Victor et al. in 1965 [5]. As per an observational study published in the European Journal of Neurology, AHD is a chronic encephalopathy that occurs in ∼1% of patients with liver cirrhosis and seems related to portosystemic shunts [6].

Despite increasing awareness, AHD remains underrecognized as a cause of cognitive impairment in patients with chronic liver disease [2]. We present a patient with the clinical and radiological features of AHD who developed rapid cognitive decline, and we discuss the possible mechanisms involved as well as currently available treatment options.

## Case presentation

A 64-year-old male with a history of poorly controlled type 2 diabetes mellitus with a glycated haemoglobin (HbA1c) of 11.7% (103 mmol/mol), obstructive sleep apnea and obesity presented to the emergency department following a mechanical fall that resulted in an acute fracture of the neck of the femur in late 2025, which was surgically fixed with a hip hemiarthroplasty. Collateral history revealed that this acute injury was the consequence of a two-year trajectory of progressive, debilitating neurological decline. 

The patient, a non-drinker and former smoker, first developed an asymmetric left-hand tremor in early 2023, which was initially diagnosed as an essential tremor but soon progressed to involve the right hand. By March 2025, he experienced episodes of confusion, aphasia, and memory loss, prompting an initial clinical workup for Parkinson's disease. He was working as a CCTV operator for the police until March when he had to stop working due to the worsening symptoms. 

His cognitive and motor functions continued to deteriorate dramatically. By mid-2025, he developed urinary incontinence, severe gait instability, and profound dyspraxia, characterized by an inability to coordinate simple movements (e.g., bringing a cup to his mouth) and the misidentification of common household objects. 

On physical examination, the patient demonstrated marked somnolence, apathy, and fluctuating levels of consciousness. Neurological assessment of the motor system revealed prominent bilateral rigidity, bradykinesia, and an ataxic gait with a pronounced tendency to lean to the left. 

Laboratory investigations demonstrated significant hepatic dysfunction. Serum ammonia was markedly elevated, peaking at 133 µmol/L (reference range <49 µmol/L), consistent with hepatic encephalopathy, and improved to 70 µmol/L following treatment with lactulose. Liver biochemistry revealed hyperbilirubinemia (total bilirubin 60 µmol/L), hypoalbuminemia (25 g/L), and a mildly prolonged international normalized ratio (INR) of 1.5, indicating impaired hepatic synthetic function. Hematological investigations demonstrated bicytopenia, comprising leukopenia and thrombocytopenia, consistent with portal hypertension and hypersplenism secondary to chronic liver disease. Glycaemic assessment showed a markedly elevated HbA1c of 103 mmol/mol, indicative of poorly controlled diabetes mellitus.

**Figure 1:**
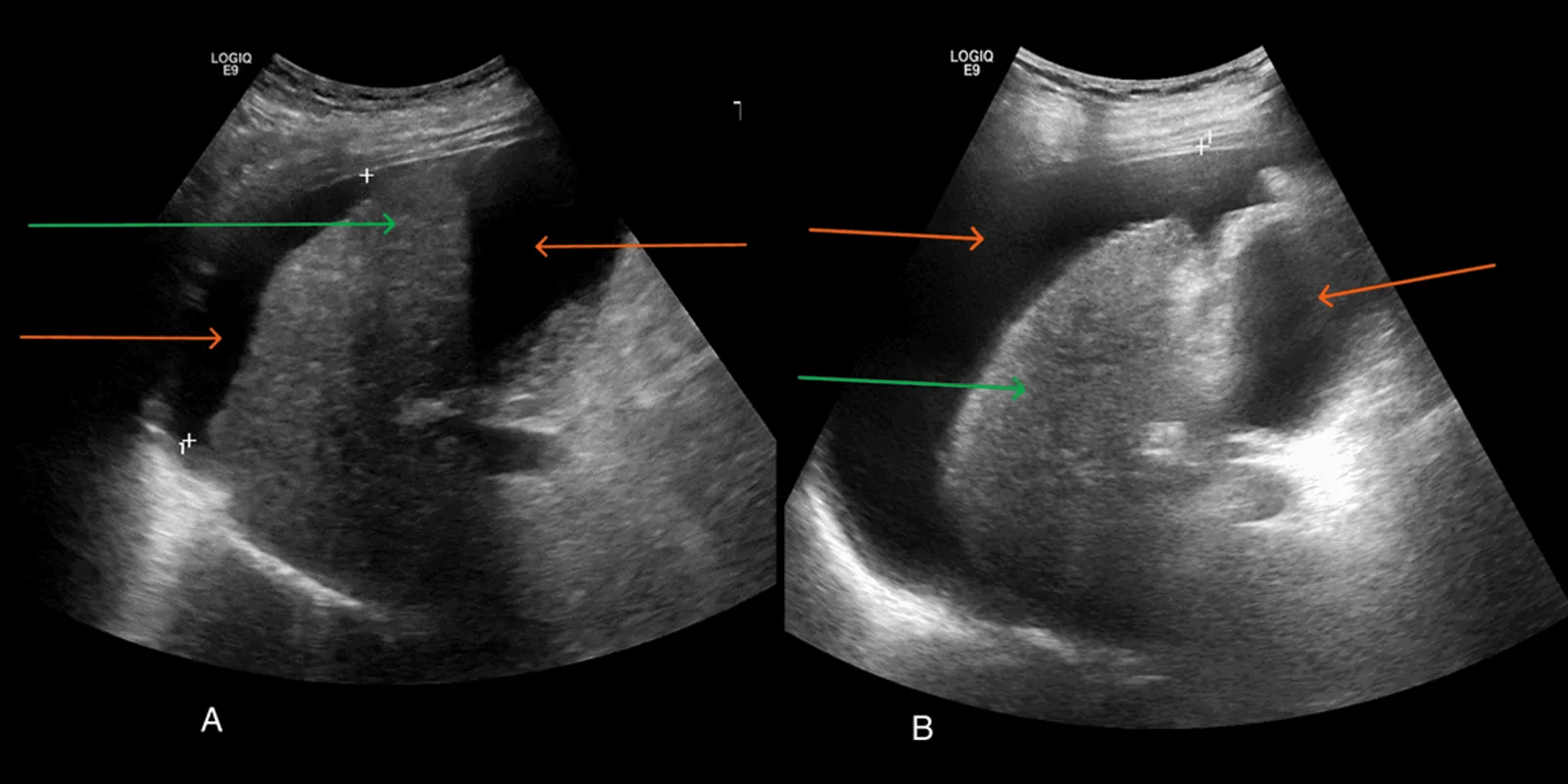
Serial abdominal ultrasound images obtained two weeks apart demonstrating progression of ascites (orange arrows). (A) Initial scan obtained on 21 October 2025 showing moderate free intraperitoneal fluid. (B) Follow-up scan obtained on 6 November 2025 demonstrating a marked increase in ascites. The echogenic liver with irregular margins is indicated by the green arrows in both panels.

Abdominal ultrasonography (Figure 1) showed features consistent with liver cirrhosis and progression of ascites over a two-week period. Based on the clinical, biochemical, and imaging findings, the patient was classified as having decompensated cirrhosis, with a Child-Pugh score of 10 (Class C) [7] and an estimated Model for End-Stage Liver Disease (MELD)-Na score of 16 [8]. Laboratory investigations during admission are presented in Table 1, while liver disease severity assessment is summarized in Table 2. 

**Table 1:** Laboratory investigations on admission INR: international normalised ratio, HbA1c: haemoglobin A1c

Parameter	Result	Reference range
Serum ammonia (µmol/L)	133	<49
Total bilirubin (µmol/L)	60	3-20
Albumin (g/L)	25	35-50
INR	1.5	0.9-1.2
White blood cell count (×10⁹/L)	4	4.0-11.0
Platelet count (×10⁹/L)	132	150-400
HbA1c (mmol/mol)	103	<48
Creatinine (µmol/L)	75	60-110
Sodium (mmol/L)	135	135-145

**Table 2:** Liver disease severity assessment MELD: Model for End-Stage Liver Disease

Score	Value	Interpretation
Child-Pugh score [7]	10	Class C (decompensated cirrhosis)
MELD-Na score [8]	16	Moderate predicted 90-day mortality
West Haven grade [9]	II	Moderate hepatic encephalopathy
Ascites	Present	Evidence of hepatic decompensation

**Figure 2:**
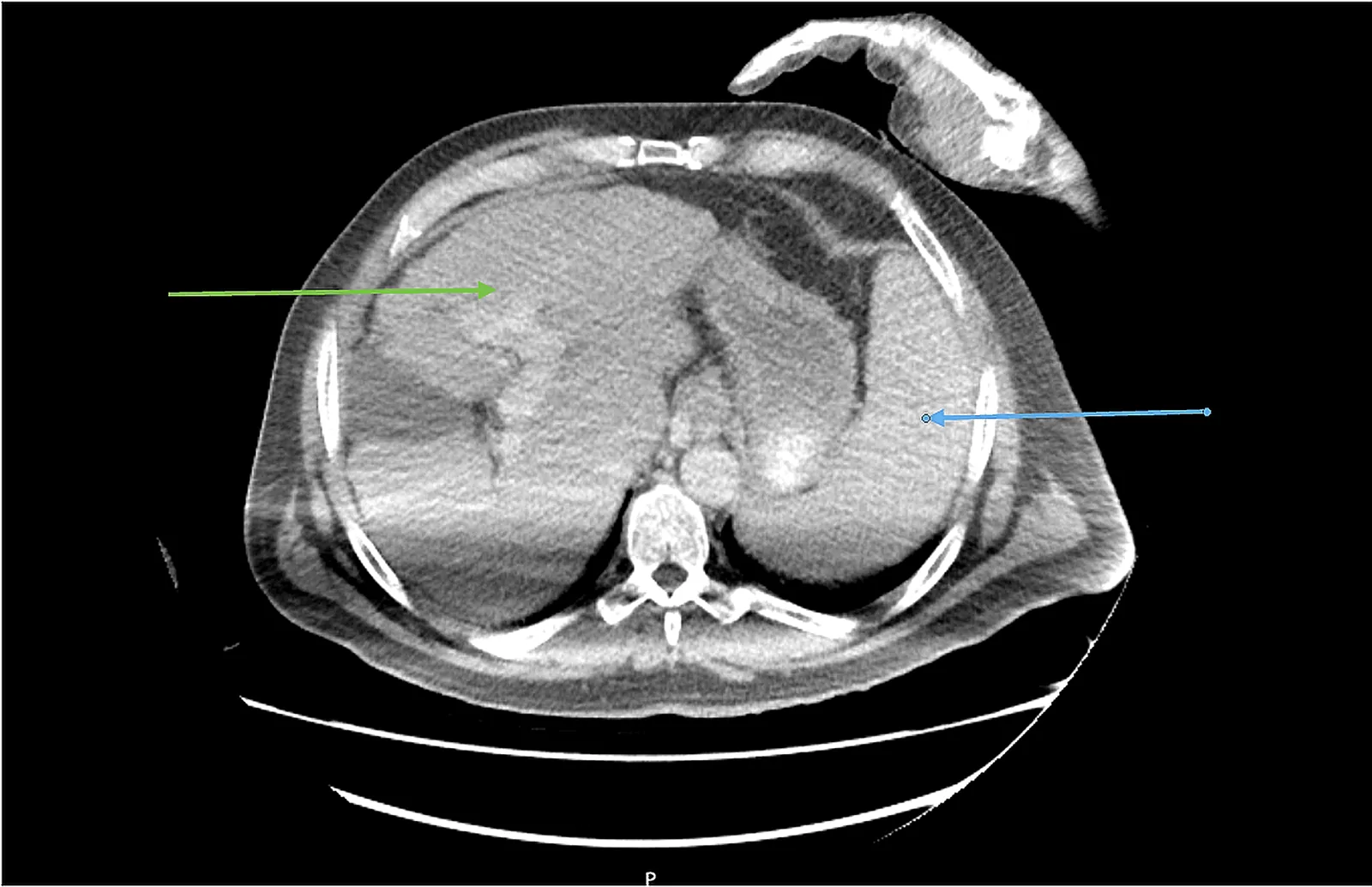
CT Abdomen and pelvis done on 16th October 2025 showing cirrhotic liver (marked with green arrow) with bulky spleen (marked with blue arrow).

Computed tomography (CT) imaging (Figure 2) demonstrated an irregular, coarse hepatic echotexture consistent with cirrhosis, accompanied by splenomegaly (136 mm), perihepatic ascites, and extensive portosystemic collateralization with antegrade portal vein flow. The underlying etiology of the liver disease was presumed to be metabolic dysfunction-associated steatotic liver disease (MASLD), driven by his severe metabolic syndrome. 

**Figure 3:**
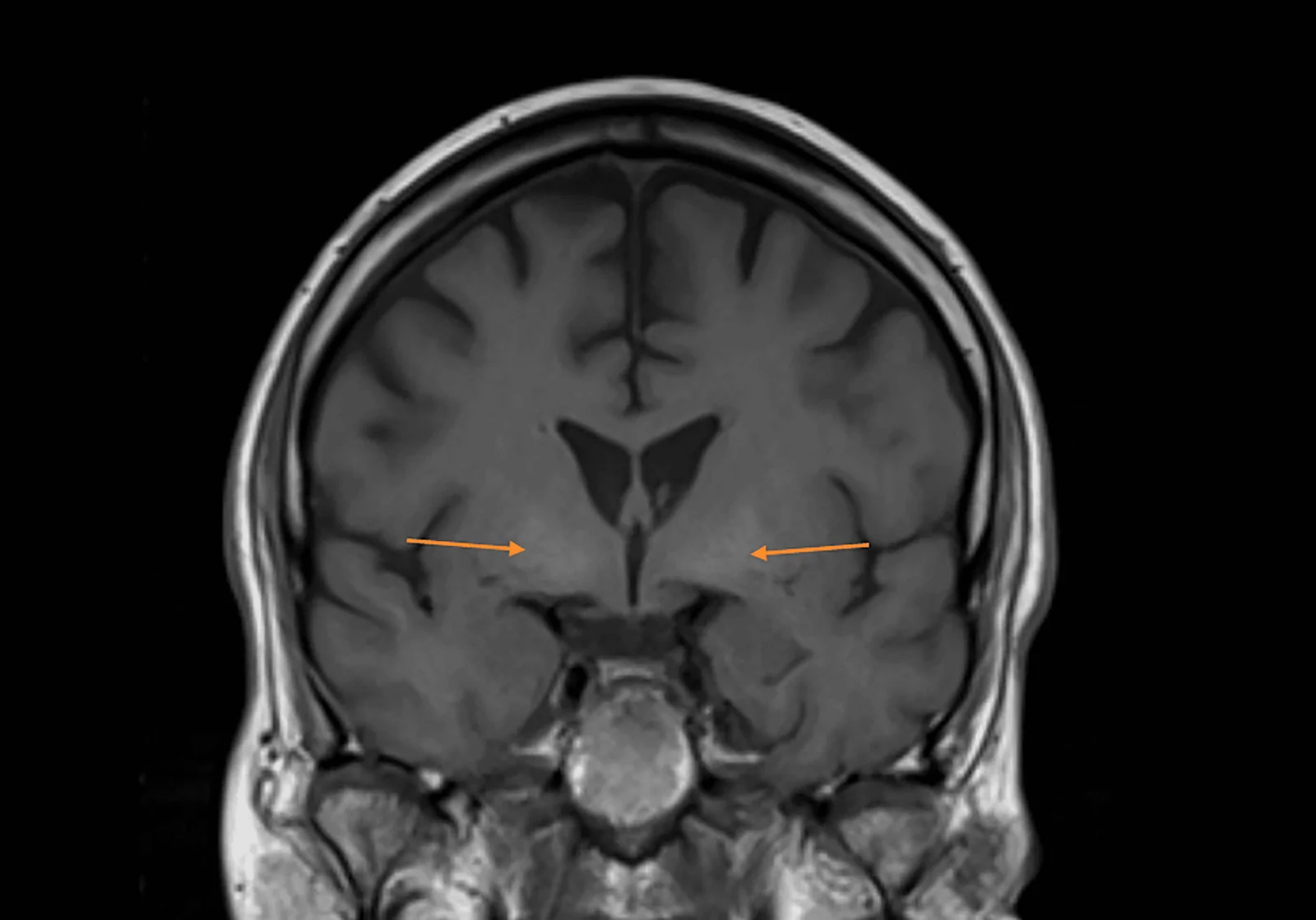
MRI Brain showing symmetrical bilateral hyper-intensities in globus pallidus (marked by orange arrow).

**Figure 4:**
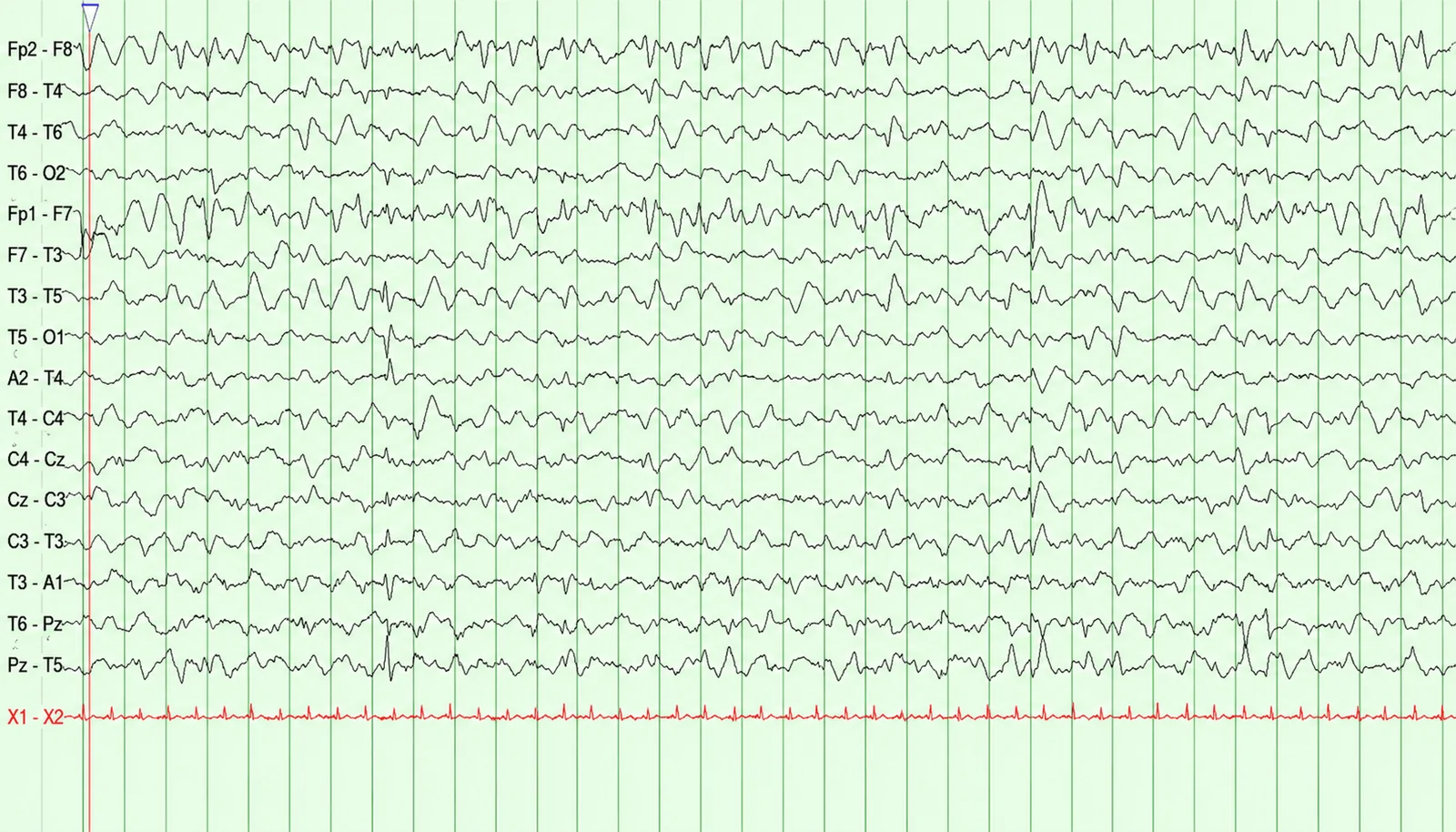
EEG showing continuous ill-defined periodic sharp wave in the anterior region throughout the recording, in keeping with encephalopathy.

A comprehensive neurological workup was undertaken to clarify the etiology of his movement disorder and cognitive decline. A retrospective review of a brain magnetic resonance imaging (MRI) scan from June 2023 revealed striking, bilateral T1-weighted hyperintensities within the basal ganglia - specifically the globus pallidus - and the brainstem (Figure 3). An electroencephalogram (EEG) demonstrated ill-defined periodic sharp wave discharges and triphasic waves at 1-1.5 Hz, consistent with a severe metabolic encephalopathy (Hepatic Encephalopathy Grade II/III) (Figure 4). 

Alternative diagnoses were excluded: Wilson's disease was ruled out by normal serum ceruloplasmin, normal 24-hour urine copper, and the absence of Kayser-Fleischer rings on slit-lamp examination; genetic testing for hereditary hemochromatosis was negative. Furthermore, his parkinsonian symptoms had shown a poor clinical response to previously prescribed levodopa therapy. MASLD was presumed to be the probable diagnosis based on steatosis on imaging, metabolic risk factors (type 2 diabetes, obesity with a BMI of 32, obstructive sleep apnea), and exclusion of viral, autoimmune, and significant alcohol‑related liver disease.

Despite adequate lactulose therapy (dose, bowel movements), the patient’s parkinsonian gait and bradykinesia remained largely unchanged, and only the fluctuating confusion improved. The episodic confusion, elevated ammonia levels, and mild improvement in mental status following lactulose therapy suggests superimposed hepatic encephalopathy.

Synthesizing the clinical triad of chronic liver disease with portosystemic shunting, an extrapyramidal movement disorder refractory to levodopa, and pathognomonic T1 MRI hyperintensities, a diagnosis of AHD was suggested by the Neurology team. The chronic, non‑fluctuating parkinsonism with cerebellar signs, together with symmetric globus pallidus T1 hyperintensity and large portosystemic collaterals, argues for AHD.

Given the advanced stage of his liver disease, with evidence of decompensation including severe hepatic encephalopathy and massive ascites, a multidisciplinary team decision was made that he was not a suitable candidate for liver transplantation. Following discussions between the gastroenterology team and the patient's family, the focus of care was shifted to best supportive management. His treatment escalation plan was also reviewed with the family, who agreed to a ward-based ceiling of care and a do-not-attempt-resuscitation (DNAR) order.

He was subsequently assessed by the palliative care team for symptom management and was discharged to a care home via a fast-track discharge pathway. He died shortly thereafter in the care home. 

The timeline has been summarized in Table 3 below.

**Table 3:** Timeline of the Patient's Clinical Course AHD: acquired hepatocerebral degeneration

Time	Clinical Events
Early 2023	Left-hand tremor developed, initially diagnosed as essential tremor.
June 2023	Brain MRI showed bilateral T1 hyperintensities in the globus pallidus and brainstem.
March 2025	Progressive confusion, aphasia, and memory impairment; evaluated for Parkinson's disease and ceased employment due to worsening symptoms.
Mid-2025	Developed bilateral rigidity, bradykinesia, ataxia, urinary incontinence, and severe dyspraxia.
Late 2025	Mechanical fall causing fractured neck of femur; admitted to hospital where previously unrecognized decompensated cirrhosis with hepatic encephalopathy was diagnosed.
Hospital admission	CT confirmed cirrhosis with portal hypertension and portosystemic shunting; EEG demonstrated hepatic encephalopathy; AHD diagnosed based on characteristic MRI findings, levodopa-refractory parkinsonism, and exclusion of alternative diagnoses.
Outcome	Not considered for liver transplantation; transitioned to palliative care and died shortly after discharge to a care home.

## Discussion

Non-Wilsonian AHD is a rare neurological complication associated with chronic liver disease and is characterised by progressive movement abnormalities, cognitive dysfunction, and neuropsychiatric manifestations. It is most frequently observed in patients with cirrhosis and portosystemic shunting and is considered pathophysiologically distinct from hepatic encephalopathy [9,10].

This case highlights the challenge of differentiating AHD from primary neurodegenerative conditions. The patient presented with tremor, bradykinesia, and cognitive decline, features that initially suggested Parkinson’s disease or an atypical parkinsonian syndrome [11]. However, the relatively rapid clinical deterioration, early onset of cognitive and behavioural symptoms, and the absence of a sustained therapeutic response to levodopa are not typical of idiopathic Parkinson’s disease [12].

An important differential diagnosis in such presentations is Wilson’s disease, particularly when movement disorders coexist with liver dysfunction. In this patient, Wilson’s disease was excluded on the basis of normal 24-hour urinary copper excretion and the absence of Kayser-Fleischer rings on slit-lamp examination. Furthermore, the patient’s age and lack of additional biochemical evidence made this diagnosis less likely [13]. Haemochromatosis was similarly excluded through genetic testing.

The diagnosis of AHD was supported by the presence of cirrhosis with portosystemic collateral vessels and characteristic radiological findings. Hyperintensity of the basal ganglia on T1-weighted MRI is a characteristic feature of AHD and is believed to reflect manganese accumulation secondary to impaired hepatic clearance and portosystemic shunting [6,9]. The combination of these imaging findings with extrapyramidal symptoms and cognitive impairment strongly favoured the diagnosis.

Although the patient experienced episodes of confusion associated with elevated ammonia levels, consistent with superimposed hepatic encephalopathy, the overall clinical course was progressive and only partially reversible. This distinction is important in clinical practice, as hepatic encephalopathy is generally fluctuating and responsive to treatment, whereas AHD represents a chronic neurodegenerative condition arising from prolonged metabolic disturbances [14,15].

The pathogenesis of AHD is still not fully understood, but metal intoxication, particularly manganese, appears to contribute to the disease [5]. Because the hepatobiliary system normally clears manganese from the blood and cerebrospinal fluid, liver dysfunction and portosystemic shunting can allow excess manganese to circulate and accumulate in the brain [16]. This buildup, especially in the basal ganglia, brainstem, cerebral cortex, and nearby white matter, is believed to cause neuronal loss, Alzheimer type 2 astrocyte changes, and reactive gliosis, ultimately producing the extrapyramidal symptoms seen in cirrhosis [17,18].

The underlying liver disease in this patient was most likely MASLD, based on the presence of obesity and poorly controlled type 2 diabetes mellitus. MASLD is now recognised as the most common cause of chronic liver disease worldwide and is closely linked to the development of cirrhosis and its associated complications, including portosystemic shunting [15]. With the increasing global burden of MASLD, cases of AHD are likely to be identified more frequently in clinical practice.

The management of AHD remains difficult. While optimisation of hepatic function and treatment of concurrent hepatic encephalopathy may lead to some symptomatic improvement, established neurological impairment is often only partially reversible. Dopaminergic medications generally provide limited benefit, a feature that helps differentiate AHD from Parkinson’s disease [12,9]. In selected patients, liver transplantation has been associated with varying degrees of neurological recovery, potentially through correction of the underlying metabolic disturbances and reduction in accumulation of neurotoxin [17]. Nevertheless, the presence of significant comorbidities, such as poorly controlled diabetes and advanced cognitive dysfunction, may restrict eligibility for transplantation [19].

This case highlights the need to consider AHD in the differential diagnosis of patients presenting with parkinsonian features and cognitive decline in the setting of chronic liver disease. Prompt recognition is important to prevent diagnostic delay or misdiagnosis and to facilitate appropriate management planning and discussions regarding prognosis.

## Conclusions

In conclusion, this case demonstrates that AHD should be considered in patients with rapidly progressive parkinsonism, cognitive decline, and atypical neuroimaging findings in the setting of chronic liver disease. In this patient, the combination of cirrhosis with portosystemic shunting, poor levodopa responsiveness, and characteristic basal ganglia T1 hyperintensities supported the diagnosis of AHD rather than primary neurodegenerative parkinsonism.

The case also emphasizes the need to look for occult liver disease, particularly MASLD-related cirrhosis, in patients with unexplained movement disorders and neurocognitive decline. Early recognition may help avoid misdiagnosis, inform prognosis, and guide appropriate multidisciplinary management, including discussion of supportive care when advanced decompensation limits definitive treatment options.
